# Post-Coronavirus Disease 2019 Triggers the Appearance of Mixed Polyneuropathy and Brain Fog: A Case Report

**DOI:** 10.3390/clinpract12030031

**Published:** 2022-04-25

**Authors:** Donají Suárez-Sánchez, Nereida Violeta Vega-Cabrera, Monserrat Fernández-Moya, Maribel Mendoza-Navarro, Ángel Bahena-Hernández, Jesús Fabian Rojas-Hernández, Librado Baños-Peña, Francisco Vladimir López-Méndez, Osmar Antonio Jaramillo-Morales

**Affiliations:** 1 Department of Medical Education and Research, Mexican Social Security Institute, Family Medicine Unit 46, Coyoacan, Mexico City 04610, Mexico; donishola@gmail.com (D.S.-S.); mendozanavarro72@gmail.com (M.M.-N.); angelbahena2005@yahoo.com (Á.B.-H.); fabia.rojas9206@gmail.com (J.F.R.-H.); libradojonas@gmail.com (L.B.-P.); vladiie_10_franck@hotmail.com (F.V.L.-M.); 2 Life Sciences Division, Nursing and Obstetrics Department, Campus Irapuato-Salamanca, University of Guanajuato, Ex Hacienda el Copal, km. 9 Carretera Irapuato- Silao, A.P. 311, Irapuato, Guanajuato 36500, Mexico; fernandez.m@ugto.mx

**Keywords:** COVID-19, sequels, neurology, diagnosis

## Abstract

Coronavirus disease 2019 (COVID-19) can directly or indirectly affect the central and peripheral nervous systems, resulting in cognitive impairment, memory problems, and a wide range of neuromuscular involvement, including neuropathies. However, the long-term neurological complications of Severe Acute Respiratory Syndrome Coronavirus 2 (SARS-CoV-2) infection are not clear. The aim this study was to analyze a case report the presence of neurological sequelae due to post-Coronavirus disease 19 in a patient without apparent previous neurological symptoms. Clinical case: A 46-year-old patient, with no relevant history for the described condition, who, after severe COVID-19 infection, started a mixed neuropathy and mental fog syndrome as the main sequel. Multiple laboratory and imaging studies were performed during and after his hospital stay, and it was corroborated by an electromyography that it occurred from a neuropathy triggered by COVID-19 infection. Conclusions: This case provides additional evidence that mixed neuropathy and brain fog syndrome are potential complications of post-coronavirus disease 2019 syndrome. The neurological sequelae that manifest after a COVID-19 episode can be rapidly enhanced as a consequence of another alteration in some systems of the organism. However, future studies are necessary to elucidate the incidence of these neurological complications, their pathophysiological mechanisms and their therapeutic options.

## 1. Introduction

The Severe Acute Respiratory Syndrome Coronavirus 2 (SARS-CoV-2) virus infection is a new disease of which little is known; its first description was in 2019, when it was said to be a potentially contagious virus, which currently continues to be an important public health problem. Worldwide since its first confirmed case in December 2019, it increase amongst most ages, thus becoming a pandemic with more than 5 million confirmed cases in Mexico, being an elementary cause for concern due to its complications and the sequelae that have occurred. The most common symptoms of Coronavirus disease 19 (COVID-19) according to the World Health Organization (WHO) Coronavirus (COVID-19) Dashboard are fever, dry cough and tiredness. Other less common symptoms that affect some patients include aches and pains, nasal congestion, headache, conjunctivitis, sore throat, diarrhea, loss of taste or smell, and skin rashes or color changes [1,2].

Most people (about 80%) recover from the disease without the need for hospital treatment. Around one in five people who contract COVID-19 develop a serious condition and experience difficulty breathing. Patients with pre-existing cardiovascular, pulmonary, metabolic, or cancerous diseases and the elderly are at increased risk of a severe course of COVID-19. However, anyone can contract COVID-19 and become seriously ill [3].

As characteristics of both the virus and the disease it causes are continually being discovered; few data exist regarding its pathogenesis, but it has drawn attention that, despite being a virus that has more affinity for lung tissue in the first instance, clinical and experimental studies have shown that the coronavirus family has a certain tropism for the central nervous system. As more is learned about the COVID-19 pandemic, it has become apparent that neurological involvement in COVID-19 may be important in some patients. Neurological symptoms have been described in patients affected by COVID-19, such as headache, dizziness, memory loss, lack of concentration, myalgia and anosmia, as well as cases of encephalopathy, encephalitis, hemorrhagic necrotizing encephalopathy, stroke, epileptic seizures and rhabdomyolysis, associated with infection by SARS-CoV-2 [4,5,6,7]. Reports have also implicated peripheral nerve damage such as mononeuropathy and a more generalized acute polyneuropathy, most often reported as Guillain–Barre syndrome. More recently, longer-term complications ‘prolonged COVID-19’ have been frequently described including fatigue, cognitive problems (especially memory loss), persistent fever, myalgia, muscle weakness, and shortness of breath (particularly on exertion). Therefore, the possible long-term neurological complications of COVID-19 infection are of great concern [8].

Knowledge of the post-acute neurological consequences of COVID-19 is still very limited. In this sense, the objective of this study was to analyze a case report of the presence of neurological sequelae due to post-COVID-19 in a patient without apparent previous neurological symptoms.

## 2. Case Presentation

A 46-year-old male patient, with the following relevant history: type 2 diabetes mellitus for 2 years under treatment with metformin and pioglitazone, high blood pressure for 6 months under treatment with losartan and hydrochlorothiazide, was studied.

He began his illness on 20 November 2020 with general malaise, a runny nose, asthenia, adynamia, fever, oppressive chest pain, receiving symptomatic treatment without improvement, increasing dyspnea, so on 5 December, he underwent a Reaction Test in Polymerase Chain for SARS-CoV-2 with a positive result; on 6 December, he presented oxygen saturation in ambient air of 63% and exacerbation of dyspnea, for which he went for medical evaluation and was immediately hospitalized.

Laboratory studies and Computed Tomography of the Thorax were carried out, establishing the following admission diagnoses: atypical multiple-focus pneumonia due to confirmed COVID-19, type I respiratory failure, uncontrolled type 2 diabetes mellitus, systemic arterial hypertension.

During his hospital stay, he required advanced management of the airway with oral endotracheal intubation for 25 days, as well as placement of a central venous catheter and arterial line, management based on antibiotics for superimposed infections and placement of a tracheostomy cannula. After removal of invasive management of the respiratory tract, the use of supplemental oxygen was continued through a tracheostomy tent with adequate saturation. So, after a few weeks of good evolution, on 31 January he was discharged with the following discharge diagnoses: septic shock of remitted pulmonary origin, atypical pneumonia of multiple foci due to COVID-19, remitted acute respiratory distress syndrome, tracheostomy status, type 2 diabetes mellitus, arterial hypertension, with treatment based on rivaroxaban 20 mg every 24 h, clarithromycin 500 mg orally every 12 h for 5 days, nebulizations with budesonide every 12 h and a supplemental oxygen river by tracheostomy cannula at 3 L per minute, as well as treatment for underlying chronic pathologies.

He was subsequently assessed by the otorhinolaryngology service on 1 March, removing the tracheostomy cannula, with review of the airway by bronchoscopy, with preservation of voice and phonation.

After his hospital discharge, he had difficulty concentrating, memory loss of recent events, moderate dyspnea, decreased muscle strength in all four limbs, difficulty walking, pain in the occipital region, radiating to the cervical region with intensity 8/10 of the visual analog scale, the symptoms persisting despite attending rehabilitation, also presenting an episode of syncope, for which new studies were carried out in March 2021:

(1) Simple computed tomography of the chest (3 March 2021): changes in the lung parenchyma probably secondary to COVID-19.
(2) Simple skull tomography (30 March 2021): where no important structural changes were observed, only a probable adenoma in the Turkish chair.
(3) Magnetic resonance of the skull with gadolinium contrast (9 April 2021): reported without structural alterations and adenoma was ruled out.
(4) Study of nerve conduction velocities, somatosensory-evoked potentials and electromyography of the lower and upper extremities (17 April 2021) that report findings compatible with post-COVID mixed polyneuropathy, evidencing the presence of a moderate to severe sensory–motor polyneuropathic process, with a mixed pattern but one which was predominantly axonal, affecting the four extremities with greater expression in the lower extremities and predominance on the right; chronic partial denervation data were recorded in the four extremities. (Table 1, Table 2 and Table 3).

In the patient’s last evaluation in September 2021, he reported decreased dyspnea, continued fatigue, short-term memory loss, lack of concentration, difficulty remembering some words, insomnia, stabbing pain in the occipital region, intensity 8/10 in the visual analog scale that radiates to the cervical region, persists with decreased strength in the four limbs, with difficulty walking, generalized painful dysesthesias predominantly in the right side of the body; this continued with physical rehabilitation and pharmacological management based on Citidin-5′ disodium monophosphate, Uridine-5 trisodium triphosphate 5 mg/3 mg one tablet every 8 h, alprazolam 0.25 mg every 24 h, pregabalin 75 mg every 24 h, requiring support to perform basic activities of daily living such as dressing, walking, grooming, and eating.

## 3. Discussion

SARS-CoV-2 mainly presents as a respiratory disease. However, a growing body of literature is emerging linking COVID-19 to injury to other organ systems, including the central and peripheral nervous systems. In this case, the clinical and neurophysiological examinations showed neurological sequelae in the patient after the COVID-19 disease. These findings reflect not only a sensorimotor axonal polyneuropathy but also the presence of brain fog, thus losing the ability to walk and sensitivity, among other consequences of this nature are memory loss, dizziness and difficulty in articulating words, difficulty in sustained speech, lack of concentration and altered cognitive abilities. These results are consistent with previous studies that have shown the presence of neurological symptoms related to COVID-19. The 10 most frequent neurological symptoms in the patients were brain fog, headache, numbness/tingling, dysgeusia, anosmia, myalgia, dizziness, pain, blurred vision and tinnitus [1,9].

Consequently, the sequelae that occur have also focused on rehabilitation, prioritizing those of the pulmonary type. In this patient, who had important risk factors for developing a severe picture of the disease, and who mainly presented respiratory symptoms, during follow-up, it was found that the greatest affectation was at the neurological level, since the studies presented that evaluated lung capacity did not show damage that corresponded to the severity of the clinical picture presented. Regarding his recently diagnosed chronic diseases (diabetes mellitus 2 and arterial hypertension), they could suggest that they play an important role in the development of sequelae, since before the disease the patient was under control and no neurological complication would have been corroborated due to chronic illness. It is important to mention that up to 30% of patients who have been recently diagnosed with a chronic disease such as diabetes mellitus already present microvascular and neurological complications, because it continues to be an underdiagnosed disease [8,10]. Therefore, we cannot exclude the possibility that the patient had neurological involvement. Therefore, knowing the temporality of these diseases and their characteristics, they were considered a fundamental part of the initial clinical symptomatology and evolution.

Therefore, that would help the theory that several neurologists have proposed, that even some of the respiratory manifestations of COVID-19 could be neuromediated, due to the neurotropism that coronaviruses have been confirmed to have and that, applying it to the clinical picture and development of the patient’s disease could justify the sequelae that he has presented. Initially he had a respiratory condition, which could be a mild neurological manifestation, taking as a principle the replication capacity of the SARS-CoV-2 virus at the level of the Central Nervous System (CNS), where the olfactory and glossopharyngeal nerves would be the entry through dissemination of the virus to the circulation and dissemination via the hematogenous route. The associated cytokine production would then increase the permeability of the blood–brain barrier to further enhance viral penetration of the CNS; the virus could also first invade peripheral nerve terminals and then enter the CNS through trans-synaptic pathways. Oral mucosa, for example, has been shown to express angiotensin-converting enzyme receptor 2, which would allow viral spread through associated cranial nerves. Another mechanism of penetration into the CNS could be through the spike protein of SARS-CoV-2, which has a sequence like neurotoxins; these small peptides and those mentioned above could be responsible for the clinical manifestations observed in patients with COVID-19 [5,6,7,8,11,12,13,14,15,16,17,18,19].

Finally, the mechanism associated with the alteration of cognition and induction of “brain fog” is not clear; however, it could be due to the high viral load in patients with COVID-19 that involves the CNS, which causes the compromise of neurons with high-level energy metabolism, causing selective mitochondrial neuronal targeting in SARS-CoV-2 infection, leading to neuroinflammation, rupture of the blood–brain barrier, microvasculitis and hypoxia, which could give rise to the symptoms of brain fog [20,21,22]. Therefore, the pathogenesis of these sequelae in COVID-19 survivors is unknown and there are many pending questions to investigate.

## 4. Conclusions

This case provides additional evidence that SARS-CoV-2 infection may have triggered or at least contributed to mixed neuropathy and brain fog syndrome worsening. The neurological sequelae that manifest after a COVID-19 episode can be rapidly enhanced as a consequence of another alteration in some systems of the organism. Therefore, when they can be detected in a timely manner, the appearance of neurological symptoms will make it easier for us to determine the severity of the infection or the possible consequences. Mortality could even be reduced, since it has already been shown that it is not only the lung condition that produces the progressive deterioration of patients. That is why, despite the fact that the respiratory manifestations are the most visible, they may not be responsible for the clinical picture and even for the post-disease state.

However, because in general in the population who suffer from some type of chronic disease, it can be difficult to know if it is a post-COVID-19 infection state with neurological sequelae or an exacerbation of a complication of chronic disease, which forces us to be more thorough in elucidating the pathophysiological mechanisms and developing and testing specific interventions for post-COVID-19 patients.

## Figures and Tables

**Table 1:** The electromyographic study shows data of chronic and active partial denervation in the indicated myotomes (low).

Side	Muscle	Nerve	Root	Ins Act	Fibs	Psw	Amp	Dur	Poly	Recrt
Left	Gastroe	Tibial	S1-2	Low	Low	+	Low	Short	2+	Inc
Left	Ext Dig Brev	Dp Br Fibular	L5, S1	Low	Nml	+	Nml	Nml	2+	Inc
Left	AntTibialis	Dp Br Fibular	L4-5	Low	Low	+	Nml	Nml	1+	Inc
Left	RectFemoris	Femoral	L2-4	Low	Low	Nml	Nml	Nml	2+	Inc
Left	1stDorInt	Ulnar	C8-T1	Nml	Nml	Nml	Nml	Nml	2+	Inc
Left	BrachioRad	Radial	C5-6	Low	Low	Nml	Nml	Nml	0	Inc
Left	Triceps	Radial	C6-7-8	Low	Low	Nml	Nml	Nml	0	Inc
Left	Biceps	Musculocut	C5-6	Low	Low	Nml	Nml	Nml	0	Inc
Left	Deltoid	Axiliary	C5-6	Low	Low	Nml	Nml	Nml	0	Inc
Right	Deltoid	Axiliary	C5-6	Low	Low	Nml	Nml	Nml	0	Inc
Right	Biceps	Musculocut	C5-6	Low	Nml	Nml	Nml	Nml	0	Inc
Right	Triceps	Radial	C6-7-8	Low	Low	Nml	Nml	Nml	0	Inc
Right	BrachioRad	Radial	C5-6	Low	Nml	Nml	Nml	Nml	0	Inc
Right	1stDorInt	Ulnar	C8-T1	Low	Low	Nml	Nml	Nml	0	Inc
Right	RectFemoris	Femoral	L2-4	Low	Low	Nml	+	Nml	2+	Inc
Right	AntTibialis	Dp Br Fibular	L4-5	Low	Low	Nml	+	Nml	2+	Inc
Right	Ext Dig Brev	Dp Br Fibular	L5, S1	Low	Nml	Nml	+	Nml	2+	Inc
Right	Gastroc	Tibial	S1-2	Low	Low	Nml	+	Nml	2+	Inc

**Table 2:** The study of peripheral, motor nerve conduction velocities shows a moderate to severe motor polyneuropathic process, with a mixed pattern but axonal predominance that affects the four extremities with greater expression in the lower extremities and predominance on the right.

Site	Latency		Amplitude		F-Lat	Segment	Distance	CV	
	(ms)	Norm	(mV)	Norm	(ms)		(cm)	(m/s)	Norm
Left Median (APB)									
Wrist	3.2	<4.2	6.6	>5.0	28.7				
Elbow	8.3	-	6.0	-		Elbow-Wrist	24	47	>50
Right Median (APB)									
Wrist	3.5	<4.2	7.7	>5.0	NR				
Elbow	8.6	-	7.5	-		Elbow-Wrist	28	55	>50
Left Ulnar (ADM)									
Wrist	2.6	<4.2	4.3	>3.0	32.7				
Bel elbow	8.6	-	4.0	-		Bel elbow-Wrist	29	48	>53
Right Ulnar (ADM)									
Wrist	2.8	<4.2	6.2	>3.0	31.6				
Bel elbow	9.2	-	4.9	-		Bel elbow-Wrist	32	50	>53
Left Fibular (EDB)									
Ankle	6.4	<6.1	0.31	>2.0	NR				
Bel Fib head	17.8	-	0.00	-		Bel fib head-Ankle	43	38	>38
Right Fibular (EDB)									
Ankle	3.4	<6.1	0.22	>2.0	NR				
Pop fossa	17.8	-	0.18	-		Pop fossa-bel fib head	-	-	>42
Left Tibial (AH)									
Ankle	5.3	<6.1	2.9	>4.4	59.8				
Knee	16.3	-	0.87	-		Knee-Ankle	44	40	>39

**Table 3:** The study of peripheral, sensory nerve conduction velocities shows a moderate to severe sensory polyneuropathic process, with a mixed pattern but axonal predominance that affects the four extremities with greater expression in the lower extremities and predominance on the right.

Site	Latency (Peak)		Amplitude (P-P)		Segment	Distance	CV	
	(ms)	Norm	(µV)	Norm		(cm)	(m/s)	Norm
Left Median								
Wrist-Dig II	3.8	<3.6	19	>10	Wrist-Dig II	14	37	>39
Righ Median								
Wrist-Dig II	3.6	<3.6	35	>10	Wrist-Dig II	14	39	>39
Left Ulnar								
Wrist-Dig V	3.8	<3.7	7	>15	Wrist-Dig V	14	37	>38
Righ Ulnar								
Wrist-Dig V	3.3	<3.7	16	>15	Wrist-Dig V	14	42	>38
Left Sural								
Calf-Lat mall	2.7	<4.0	17	>5	Calf-Lat mall	14	52	>35
Right Sural								
Calf-Lat mall	2.4	<4.0	13	>5	Calf-Lat mall	14	58	>35

## Data Availability

Data supporting the findings are available from the corresponding author upon reasonable request.
